# HIV-1 *pol* gene diversity and molecular dating of subtype C from Sri Lanka

**DOI:** 10.1371/journal.pone.0234133

**Published:** 2020-06-11

**Authors:** Ajit Patil, Jayanthi P. Elwitigala, Lilani Rajapaksa, Raman Gangakhedkar, Devidas Chaturbhuj, Razia Pendse, Dharshani Iruka Rajapaksha, B. B. Rewari, Nilmini Malliawadu, Kanchana Jayamanna, Dammika Dombawela, Swarali Kurle

**Affiliations:** 1 HIV Drug Resistance Laboratory, National AIDS Research Institute, Pune, India; 2 National STD/AIDS Control Programme, Colombo, Sri Lanka; 3 Indian Council of Medical Research, New Delhi, India; 4 Department of Microbiology and Immunology, Weill Cornell Medical College, New York, New York, United States of America; 5 World Health Organization, Colombo, Sri Lanka; 6 World Health Organization South-East Asia Region, New Delhi, India; Consejo Superior de Investigaciones Cientificas, SPAIN

## Abstract

**Background:**

The first case of HIV infection in Sri Lanka was reported in 1987 and at the end of 2018 there were 3500 people living with HIV. There have been commendable efforts made towards the detection, treatment, and prevention of HIV in the country. Even though the genetic diversity of HIV has been shown to affect the parameters ranging from detection to vaccine development, there is no data available with respect to the molecular epidemiology of HIV-1 in Sri Lanka.

**Methods:**

In this report we have performed the ancillary analysis of *pol* gene region sequences (n = 85) obtained primarily for the purpose of HIV-1 drug resistance genotyping. Briefly, dried blood spot specimens (DBS) collected from HIV-1 infected individuals between December 2015 and August 2018 were subjected to *pol* gene amplification and sequencing. These *pol* gene sequences were used to interpret the drug resistance mutation profiles. Further, sequences were subjected to HIV-1 subtyping using REGA 3.0, COMET, jPHMM and, RIP online subtyping tools. Moreover, Bayesian phylogenetic analysis was employed to estimate the evolutionary history of HIV-1 subtype C in Sri Lanka.

**Results:**

Our analysis revealed that the majority (51.8%) of *pol* gene sequences were subtype C. Other than subtype C, there were sequences categorized as subtypes A1, B, D and G. In addition to pure subtypes there were sequences which were observed to be circulating recombinant forms (CRFs) and a few of the recombinants were identified as potential unique recombinants (URFs). We also observed the presence of drug resistance mutations in 56 (65.9%) out of 85 sequences. Estimates of the Bayesian evolutionary analysis suggested that the HIV-1 subtype C was introduced to Sri Lanka during the early 1970s (1972.8).

**Conclusion:**

The findings presented here indicate the presence of multiple HIV-1 subtypes and the prevalence of drug resistance mutations in Sri Lanka. The majority of the sequences were subtype C, having their most recent common ancestor traced back to the early 1970s. Continuous molecular surveillance of HIV-1 molecular epidemiology will be crucial to keep track of drug resistance, genetic diversity, and evolutionary history of HIV-1 in Sri Lanka.

## Introduction

Sri Lanka, with its 9 provinces and 25 districts, accommodates a population of 21.8 million [1]. The latest HIV epidemic estimates for Sri Lanka, as per The Joint United Nations Programme on HIV/AIDS (UNAIDS) are 3500 (3100–4000) people living with HIV (PLHIVs) and < 0.1% HIV prevalence among the adult population between 15–49 years [2]. HIV epidemic in Sri Lanka is mainly attributed to six key populations (KP)–female sex workers (FSW), people who inject drugs (PWID), men who have sex with men (MSM), transgender women (TGW) and beach boys (BB). Between 2013–2018 male-to-female and male-to-male sexual transmissions are observed to be the most frequent modes of HIV transmission. On the contrary, HIV transmissions have shown to be very negligible due to injecting drug use and mother to child transmission [3].

According to the recent statistics by the National STD/AIDS Control Programme (NSACP), Sri Lanka, the HIV epidemic, which was mostly concentrated in the Western and North-Western provinces of Sri Lanka has expanded to the Southern province and some districts in North-Central and Northern provinces. Colombo and Gampaha districts are the most affected areas from 2016–2018 [3]. The United Nations (UN) aims to end HIV/AIDS by 2030 via its 90-90-90 programme. The 90-90-90 narrative works on the target of a) 90% of all PLHIVs will know their HIV status by 2020; b) 90% of all people diagnosed with HIV will receive continued antiretroviral therapy (ART) by 2020; and c) 90% of all people receiving ART will have viral suppression [4]. Sri Lanka has set the ambitious target of ending AIDS by 2025. According to the latest data, Sri Lanka stands at 77-58-85 at the end of 2018 while marching towards the 90-90-90 target, emphasizing the need for improvement in HIV testing and treatment strategies [3].

Sri Lanka introduced ART for prevention of mother to child transmission (PMTCT) in 2002. Subsequently, free ART was made available to all PLHIVs from 2004 onwards, and by the end of 2018 the number of PLHIVs receiving an ART was 1574. At the onset of the free ART programme the first-line regimen mainly consisted of the zidovudine (AZT) based regimens, zidovudine + lamivudine + efavirenz (AZT+3TC+EFV) and an alternative regimen, zidovudine + lamivudine + nevirapine (AZT+3TC+NVP). Tenofovir has been incorporated into the ART regimens since 2013. Boosted protease inhibitor plus two NRTIs zidovudine + lamivudine + ritonavir boosted-atazanavir or lopinavir (AZT+3TC+ ATV/r or LPV/r) and tenofovir + lamivudine/emtricitabine + ritonavir-boosted atazanavir or lopinavir (TDF+3TC/FTC+ ATV/r or LPV/r) is the recommended second-line regimen for adults, adolescents and children. According to NSACP 2018 statistics, out of 1574 receiving ART, 1499 were on the first-line regimen and the remaining 75 were on second-line regimen. [3, 5–7].

There is enough information available regarding the incidence and prevalence of HIV, the rate of new HIV infections and deaths occurring due to AIDS, the number of people receiving ART, and people with ART showing suppressed viral loads [2, 3]. There is also enough data available with respect to, most at risk key population within the country [8, 9]. Unfortunately, with respect to the Sri Lankan HIV epidemic, there is no literature available elucidating the genetic makeup of HIV in Sri Lanka. Moreover, there are no HIV sequences available in the Los Alamos HIV sequence database (https://www.hiv.lanl.gov/) from this part of the world.

HIV genetic diversity has been directly linked to viral diagnosis, drug resistance development, and response to ART. HIV-1 diversity across the globe is continuously evolving and understanding the complex nature of global HIV-1 molecular epidemiology is key to HIV vaccine development [10]. In this report, we are presenting for the first time the data on HIV-1 subtypes and CRFs in Sri Lanka using *pol* gene sequences. NSACP, Sri Lanka, is supported by the National Reference Laboratory (NRL) for the routine HIV diagnostic activities. The HIV drug resistance laboratory at the Indian Council of Medical Research-National AIDS Research Institute, India (ICMR-NARI), has been actively engaged with the NSACP and NRL, Sri Lanka, for HIV drug resistance genotyping since March 2015. The purpose of this collaborative activity is to provide the necessary support to NSACP, Sri Lanka, for better decision making towards patient clinical management. Through this collaborative activity, ICMR-NARI has been receiving the dried blood spot specimens (DBS) from NSACP, Sri Lanka, for HIV-1 drug resistance genotyping.

HIV-1 *pol* gene sequences generated for HIV-1 drug resistance genotyping were obtained using DBS specimens (n = 85) collected between December 2015 and August 2018. The emphasis of the analysis performed in this report is to explore the subtype diversity and evolutionary history of HIV-1 in Sri Lanka. However, a limitation of this report lies in the fact that, the findings presented here are based on the limited region of the HIV-1 genome and must be substantiated further using full-length genome or multiple gene region sequences.

## Materials and methods

### Study population

DBS specimens were collected from HIV-1 infected individuals registered under the NSACP, Sri Lanka ART program. DBS specimens used in this study were collected between December 2015 and August 2018. Specimens were shipped to ICMR-NARI, India for further processing. Since the individuals are registered under the national ART program, no consent was taken for sample collection. The manuscript is based on the secondary analysis of the sequence data and the same was approved by the Ethics Committee, National AIDS Research Institute, Pune, India (Ref: NARI/EC/Approval/2019/264).

### Amplification and sequencing of HIV-1 *pol* region

DBS specimens were subjected to total nucleic acid (TNA) extraction using the NucliSens EasyMag total nucleic acid extraction system (Biomerieux). HIV-1 *pol* region encompassing protease codons 1–99 and reverse transcriptase codons 1–345 was amplified using the set of primers **(S1 Table)** mentioned in the WHO manual for HIV drug resistance testing using dried blood spot specimens [11]. Briefly, TNA extracted was subjected to reverse transcription and first-round amplification using QIAGEN one step-RT PCR kit (QIAGEN) using primer sets PR1-PR2 and RT1-RT2. The first-round product was subjected to nested amplification using AmpliTaq Gold DNA polymerase (Thermo Fisher Scientific) and primer sets PR3-PR4 and RT3-RT4. Amplified PCR products were subjected to cycle sequencing using the Big Dye Terminator v3.1cycle sequencing kit (Thermo Fisher Scientific). Applied Biosystems 3130XL Genetic Analyzer (Thermo Fisher Scientific) was used to collect the sequencing data. Contig generation and sequence base calling was performed using SeqScape v2.6 software (Thermo Fisher Scientific). Sequence FASTA files generated were employed for further analysis.

### HIV-1 subtyping

For subtyping analysis, the sequences generated (n = 85) were aligned and trimmed using MEGA 6 [12]. These sequences with equal length were encompassing the HXB2 coordinates 2281–3358. HIV-1 subtyping was performed with the help of REGA HIV-1 Subtyping Tool version 3.0 [http://dbpartners.stanford.edu:8080/RegaSubtyping/stanford-hiv/typingtool/] [13], COMET HIV-1 (COntext-based Modeling for Expeditious Typing) [https://comet.lih.lu/] [14], jpHMM-HIV [http://jphmm.gobics.de/submission_hiv] [15] and RIP (Recombinant Identification Program) [https://www.hiv.lanl.gov/content/sequence/RIP/RIP.html] [16].

For a given sequence, the subtype was directly assigned when at least three out of four subtyping tools used showed concordance. Two sequences that showed a discrepancy in subtyping results, were submitted to BLAST (Basic Local Alignment Search Tool) [https://blast.ncbi.nlm.nih.gov/Blast] to find the most closely related sequences and to confirm the subtype of these sequences.

A maximum likelihood (ML) tree was constructed with the help of IQ-Tree program [https://www.hiv.lanl.gov/content/sequence/IQTREE/iqtree.html] [17]. Briefly, the sequences were submitted to the IQ-Tree platform and the phylogenetic tree was constructed using a general time- reversible plus gamma (GTR+G) nucleotide substitution model with 1000 bootstrap replicates using an ultrafast bootstrap mode. The Newick file for the ML tree generated was further visualized and edited either with the help of MEGA 6 or FigTree v1.4.2.

For the depiction of recombinants, recombination breakpoint data obtained using jpHMM-HIV was used to map the breakpoints on the HXB2 genome with the help of Recombinant HIV-1 Drawing Tool (https://www.hiv.lanl.gov/content/sequence/DRAW_CRF/recom_mapper.html).

### Drug resistance mutation analysis

For HIV-1 drug resistance mutation interpretation, sequences were analyzed with the help of the HIVdb Program, Genotypic Resistance Interpretation Algorithm [https://hivdb.stanford.edu/hivdb/by-sequences/] available at Stanford University HIV Drug Resistance Database [18]

### Dataset for Bayesian analysis

Bayesian evolutionary analysis was performed for Sri Lankan subtype C sequences. For this, reference sequences closely related (≥ 95% bootstrap support) to Sri Lankan HIV-1 subtype C sequences were retrieved with the help of the ViroBLAST tool using 44 Sri Lankan subtype C sequences as query [19]. 136 reference sequences were retained after duplicate sequences were removed and only one sequence per patient having country and sampling date information were retained for analysis. These reference sequences included sequences from Burundi (n = 2), Botswana (n = 5), Switzerland (n = 2), China (n = 5), Denmark (n = 1), Ethiopia (n = 2), Great Britain (n = 3), India (n = 81), Italy (n = 1), Kuwait (n = 5), Malawi (n = 1), Nepal (n = 4), Sweden (n = 1), Tanzania (n = 1), Uganda (n = 1), USA (n = 5), Yemen (n = 1), South Africa (n = 5), Zambia (n = 8) and Zimbabwe (n = 2).

These reference sequences (n = 136) were combined with the 44 Sri Lankan subtype C sequences. To examine the clustering of Sri Lankan sequences with these reference sequences, a ML tree was constructed as explained earlier. As shown in **Fig 4**, the majority of the reference sequences were composed of isolates from India and most of the Sri Lankan subtype C sequences (n = 34) were clustering with Indian subtype C sequences. Within this cluster of Indian and Sri Lankan sequences, there were 21 Sri Lankan sequences forming a monophyletic cluster. There were 10 Sri Lankan sequences that were clustering with the group of sequences from Africa, USA, Great Britain, Kuwait, and sequences of European origin.

Only sequences present in this India-Sri Lanka sequence cluster (sequences with blue taxon names) were selected for further analysis. This cluster contained a total of 109 sequences, from Sri Lanka (n = 34), India (n = 67), Nepal (n = 4), and China (n = 4). Before commencing further analysis, major drug resistance sites were removed from the sequences. Temporal signal for molecular clock construction in this dataset was checked by TempEst v1.5 [20] and incongruent sequences (n = 6) were excluded. The final dataset consisted of a total of 103 sequences, from Sri Lanka (n = 34), India (n = 63), Nepal (n = 4), and China (n = 2).

### Bayesian maximum clade credibility tree and phylodynamic analysis

A dataset containing 103 sequences, from Sri Lanka (n = 34), India (n = 63), Nepal (n = 4), and China (n = 2) was used for the construction of a time-scaled maximum clade credibility tree. As noted earlier, all major drug resistance mutation sites were excluded from this dataset. The best-fitting substitution model for the dataset used was a general time-reversible substitution model with a gamma-distributed rate of variation and a proportion of invariant sites (GTR+G+I), as determined by jModelTest 2.1 [21]. A Bayesian MCC tree was constructed using BEAST v1.10.4 [22] with BEAGLE library [23]. BEAST analysis was carried out using an uncorrelated relaxed lognormal parameter with a GTR+G+I substitution model and Bayesian skyline plot analysis to estimate the effective population size [24]. During the BEAST analysis, tMRCA estimation and Bayesian Skyline reconstruction was restricted to the taxon set constituting a monophyletic cluster of 21 Sri Lankan subtype C sequences. The BEAST analysis was performed for 100 million steps with sampling at every 10000 states. Outputs were examined using Tracer v 1.7.1 with a 10% burn-in to make sure that all the parameters have an effective sampling size (ESS) of more than 200. The final annotated MCC tree was generated using Tree Annotator v1.10.4 with the burn-in of 10% and the annotated tree was visualized using FigTree v1.4.2.

### Sequence data

Nucleotide sequences were submitted to GenBank. The accession numbers for these sequences are MN443788—MN443872.

## Results

### Demographic characteristics of patients

These individuals were from 18 different districts of Sri Lanka, a majority of whom were from the Gampaha district (n = 19), followed by 11 individuals from Colombo **(S2 Table)**. According to NSACP, Sri Lanka, a noteworthy fraction of PLHIVs in Sri Lanka have a history of external migration [3]. Demographic information collected for these patients revealed that 26 out of 85 patients had a travel history outside of Sri Lanka, notably for employment, and 19 out of these 26 individuals informed about their involvement in casual sex, while none of them had any account of blood transfusion or intravenous drug uses. Out of 85 individuals, 46 were male and 39 were female, and the median age for these individuals was 40 years. All these individuals were enrolled under the NSACP, Sri Lanka ART program. ART status of 78 individuals was known while that of the remaining 7 was not available. Out of these78 individuals, the majority (n = 52) were receiving a TDF based therapy. For most of these individuals DBS specimens were collected for HIV drug resistance genotyping after confirming a virologic failure by performing a viral load estimation.

### HIV-1 *pol* gene subtyping

A total of 85 sequences spanning HXB2 coordinates 2281–3358 (1079 bp) were subjected to subtype analysis (S3 Table). Subtype assignment was done using REGA 3.0, COMET, jPHMM, and RIP subtyping tools. For a given sequence the subtype was assigned when at least three out four subtyping tools showed concordance. Out of 85 sequences 66 (77.64%) showed the complete concordance for subtype assignment using REGA 3.0, COMET, jPHMM, and RIP subtype assignment tools, while for 17 (20.0%) sequences, 3 out of four tools showed the concordance. Two (2.36%) sequences IDM35_17–11331 and IDF576_15–10720, showed a discrepancy in the assignment of subtype. For sequence IDM35_17–11331, REGA and COMET assigned the subtype CRF02_AG, while jPHMM and RIP assigned subtype A1G and G, respectively. IDF576_15–10720 was assigned subtype G by REGA while COMET was not able to assign any subtype. A1G and G subtypes were assigned by jPHMM and RIP respectively for IDF576_15–10720. Ultimately, these sequences were subjected to BLAST analysis to search for similar sequences. After performing the BLAST search, IDM35_17–11331 was confirmed to be CRF02_AG and IDF576_15–10720 was assigned to subtype G.

**Fig 1:**
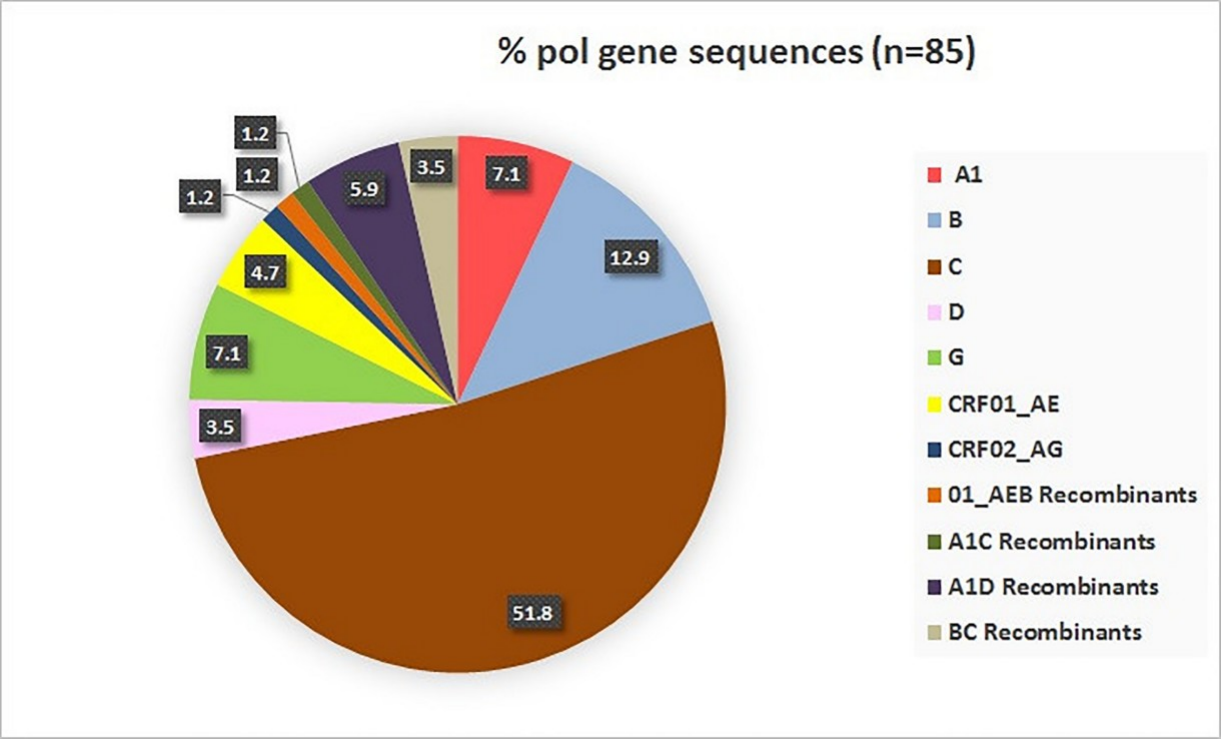
HIV-1 subtypes. The pie chart illustrates the proportion (%) of various HIV-1 subtypes based on the *pol* gene sequences (n = 85). Data labels indicate the individual proportion of each subtype.

As shown in **Fig 1**, out of 85 sequences, 44 (51.8%) were assigned to subtype C by all the subtyping tools. Subtype B was the next predominant subtype, with a total of 11 (12.9%) sequences categorized as subtype B. Along with subtype C and B sequences, there were 6 (7.1%) sequences belonging to HIV-1 subtype A1, while 6 (7.1%) sequences were of subtype G and 3 (3.5%) sequences were assigned to subtype D.

Other than pure subtypes, there were 15 (17.7%) sequences that were classified as the recombinant forms. Out of these 15 recombinant forms, 4 (4.7%) were assigned to the CRF01_AE subtype by all the four subtyping tools used. There were 5 (5.9%) sequences that were categorized as the A1D recombinants, while the numbers of CRF_02AG and A1C recombinants were 1(1.2%) and 1 (1.2%) respectively. There were 3 (3.5%) BC recombinants and one (1.2%) sequence was observed to be CRF01_AEB recombinant.

**Fig 2:**
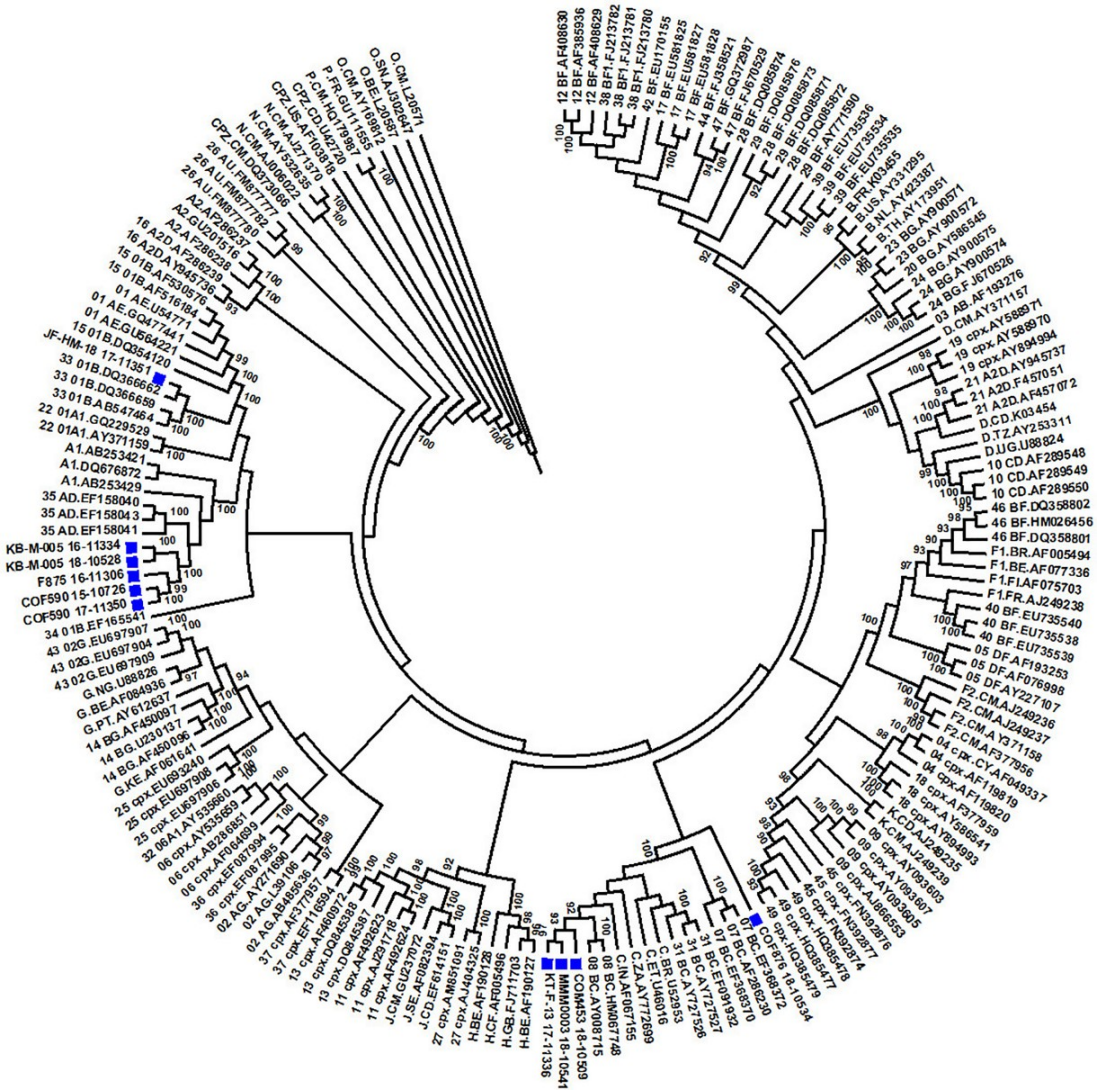
Maximum likelihood tree for Sri Lankan HIV-1 recombinant sequences (MMM0003_18–10541, KT-F-13_17–11336, COM453_18–10509, KB-M-005_18–10528, KB-M-005_16–11334, F875_16–11306, COF590_17–11350, COF590_15–10726, COF876_18–10534 and JF-HM-18_17–11351). Tips with solid blue square are the sequences from Sri Lanka. Only bootstrap values≥ 90% are displayed in the tree.

A maximum likelihood tree (**Fig 2**) was constructed for 10 recombinant Sri Lankan HIV-1 sequences, which were not assigned any existing CRF nomenclature. Reference sequences were obtained from the Los Alamos HIV sequence database (https://www.hiv.lanl.gov/content/sequence/NEWALIGN/align.html). Sequences identified as BC recombinants (MMM0003_18–10541, KT-F-13_17–11336 and COM453_18–10509) were clustering with CRF08_BC reference sequences. Five sequences (KB-M-005_18–10528, KB-M-005_16–11334, F875_16–11306, COF590_17–11350 and COF590_15–10726) which were subtyped as A1D recombinants clustered with CRF35_AD sequences. One A1C recombinant sequence (COF876_18–10534) was found to be grouping with a cluster of sequences containing subtype C and BC recombinants, although this A1C recombinant was observed to be segregating away from the subtype C and BC sequence cluster. Sequence JF-HM-18_17–11351, classified as a CRF01_AEB recombinant, was clustering tightly with CRF33_01B reference sequences.

**Fig 3:**
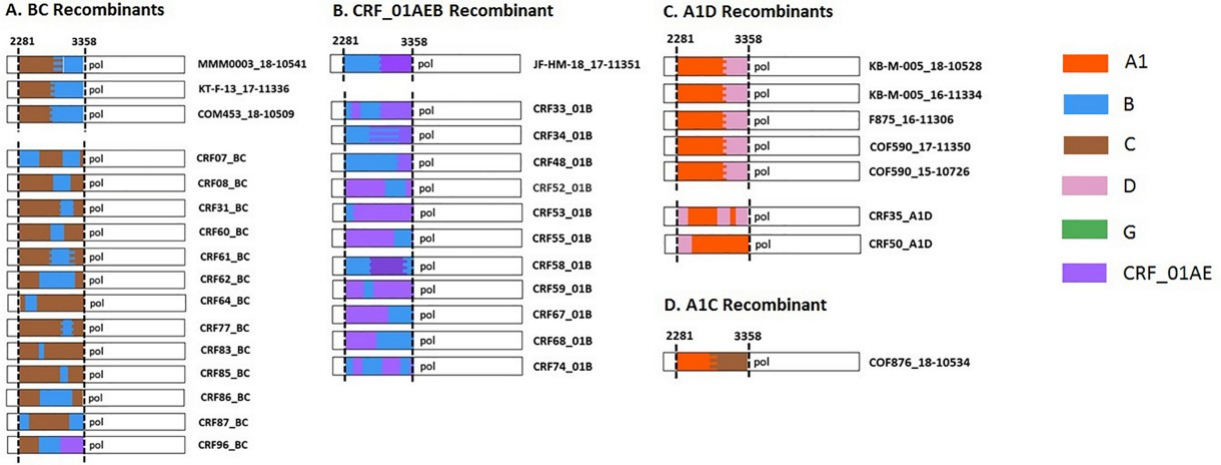
Recombination pattern of potential URFs (A-D). Comparison of recombination breakpoints between Sri Lankan recombinant sequences (top panel sequences with specimen identifiers) and respective reference CRFs (bottom panel sequences labelled with CRF identifiers) having evidence of recombination between respective subtypes within HXB2 coordinates 2281–3358. There was no reported CRF having recombination between subtypes A1 and C within the given coordinates as shown for COF876_18–10534 (D).

Further, these recombinants were examined for their recombination pattern resemblance to CRFs having evidence of recombination within HXB2 coordinates 2281–3358. **Fig 3**, shows that all of these recombinant sequences were differing in their recombination breakpoints compared to respective references (https://www.hiv.lanl.gov/content/sequence/HIV/CRFs/CRFs.html) having evidence of recombination within the given region. Moreover, there is no known CRF with recombination of subtypes A1 and C in the given region as observed for COF876_18–10534. These observations point to the fact that even though these recombinants are clustering with respective well established CRFs in the phylogenetic tree, their recombination patterns differ from these reference sequences, highlighting the possibility of these 10 sequences being potential URFs.

Overall, the major HIV-1 subtype identified for Sri Lankan sequences was subtype C. Other than subtype C there were sequences belonging to subtypes A1, B, D, and G. The recombinant forms identified included the CRF01_AE, CRF02_AG and potential URFs.

### Drug resistance mutations

All these patients (n = 85) were receiving ART and for almost all of them the HIV-1 drug resistance genotyping was done after the detection of virologic failure. As shown in Table 1, we detected the presence of drug resistance mutations (DRM) in 56 (65.9%) out of 85 sequences, while 29 (34.1%) sequences had no DRMs in either protease or reverse transcriptase segment. There was only one (1.2%) sequence that had a major DRM against protease inhibitors (PIs). There were 37 (43.5%) sequences that had the DRMs against nucleoside reverse transcriptase inhibitors (NRTIs) while 50 (58.8%) sequences had the DRMs against non-nucleoside reverse transcriptase inhibitors (NNRTIs). Amongst, these sequences harboring DRMs against NRTIs and NNRTIs, there were 31 (36.5%) sequences which had the DRMs against both the class of drugs.

**Table 1:** Frequency of drug resistance mutations observed in *pol* gene sequences (N = 85) obtained from patients in Sri Lanka.

DRM	N (%)
Sequences with DRM	56 (65.9)
Sequences with no DRM	29 (34.1)
DRM against PIs	1 (1.2)
DRM against NRTIs	37 (43.5)
DRM against NNRTIs	50 (58.8)
DRM against NRTIs and NNRTIs	31 (36.5)

Within the sequences having mutations against NRTIs, M184V mutation was the most frequent, with 31 sequences out of 37. Out of 50 sequences having DRMs against NNRTIs, K103N mutation was present in the majority of them, with 29 sequences possessing this mutation.

### Time-scaled MCC tree and population dynamics for HIV-1 subtype C in Sri Lanka

**Fig 4:**
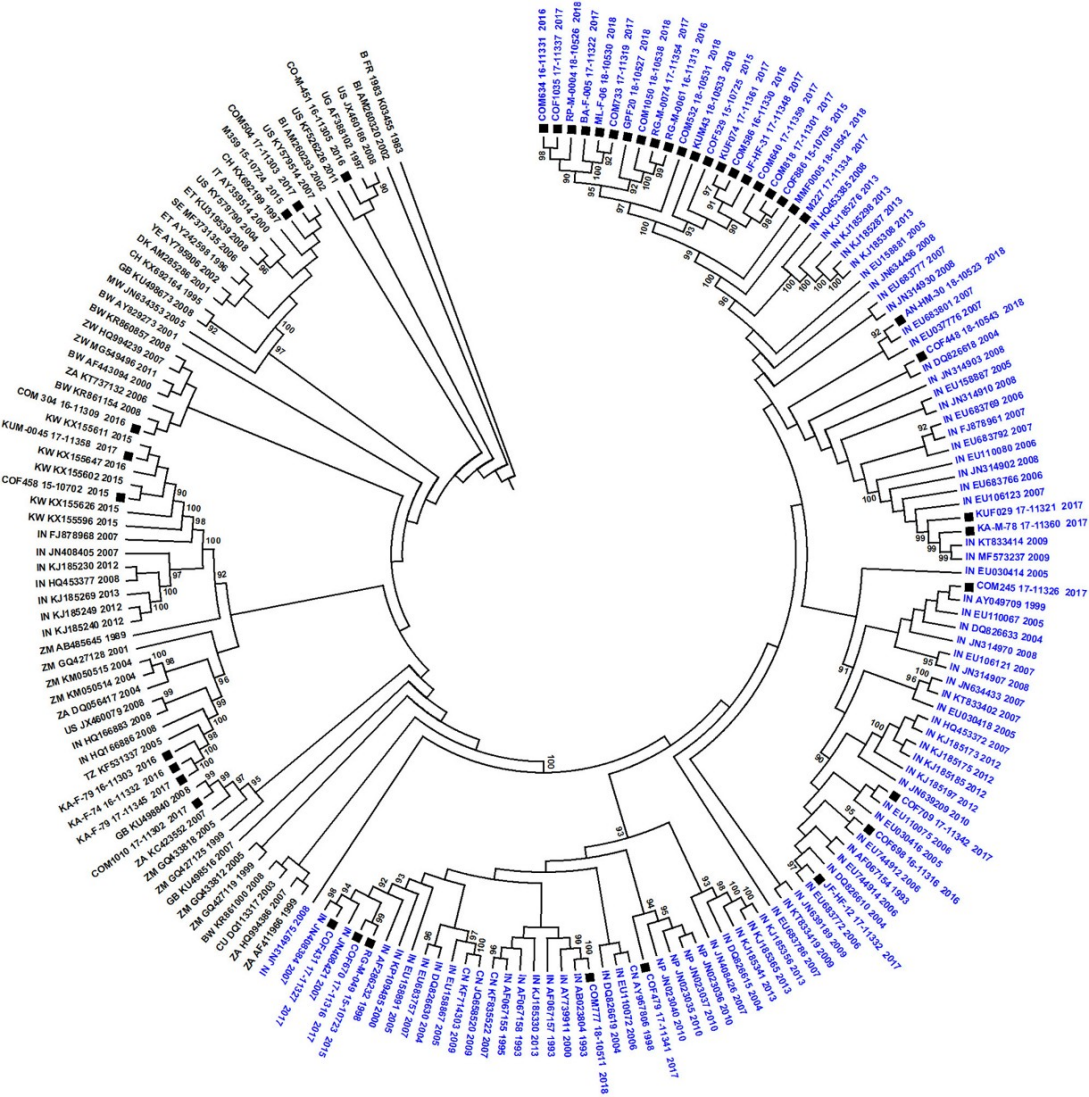
Maximum Likelihood tree showing the clustering of Sri Lankan HIV-1 subtype C sequences with closely related reference sequences. Tips with solid black square are the sequences from Sri Lanka. The first two letters in the reference taxon names represent the geographical locations of the sequence (BI = Burundi, BW = Botswana, CH = Switzerland, CN = China, CU = Cuba, DK = Denmark, ET = Ethiopia, GB = Great Britain, IN = India, IT = Italy, KW = Kuwait, MW = Malawi, NP = Nepal, SE = Sweden, TZ = Tanzania, UG = Uganda, US = United States of America, YE = Yemen, ZA = South Africa, ZM = Zambia and ZW = Zimbabwe). Only bootstrap values ≥ 90% are displayed in the tree. Sequences with a blue taxon name represents the dataset used for further analysis.

Sri Lankan subtype C sequences were subjected to Bayesian analysis for the construction of a time-scaled MCC tree. Briefly, the sequences most closely related to the Sri Lankan HIV-1 subtype C sequences were obtained as described in the methods. As shown in Fig 4, most of the Sri Lankan sequences were tightly clustering with the sequences from India. Only sequences present in the India-Sri Lanka cluster (sequences with blue taxon names) were considered for the construction of the MCC tree. Thus, the final dataset for this analysis consisted of a total of 103 sequences, from Sri Lanka (n = 34), India (n = 63), Nepal (n = 4), and China (n = 2). As noticed with TempEst v1.5, this dataset had enough temporal signal for molecular clock construction (correlation coefficient = 0.59, r^2^ = 0.34). Using this dataset, the time-scaled MCC tree was constructed to estimate the recent evolutionary history of HIV-1 subtype C introduction in Sri Lanka.

**Fig 5:**
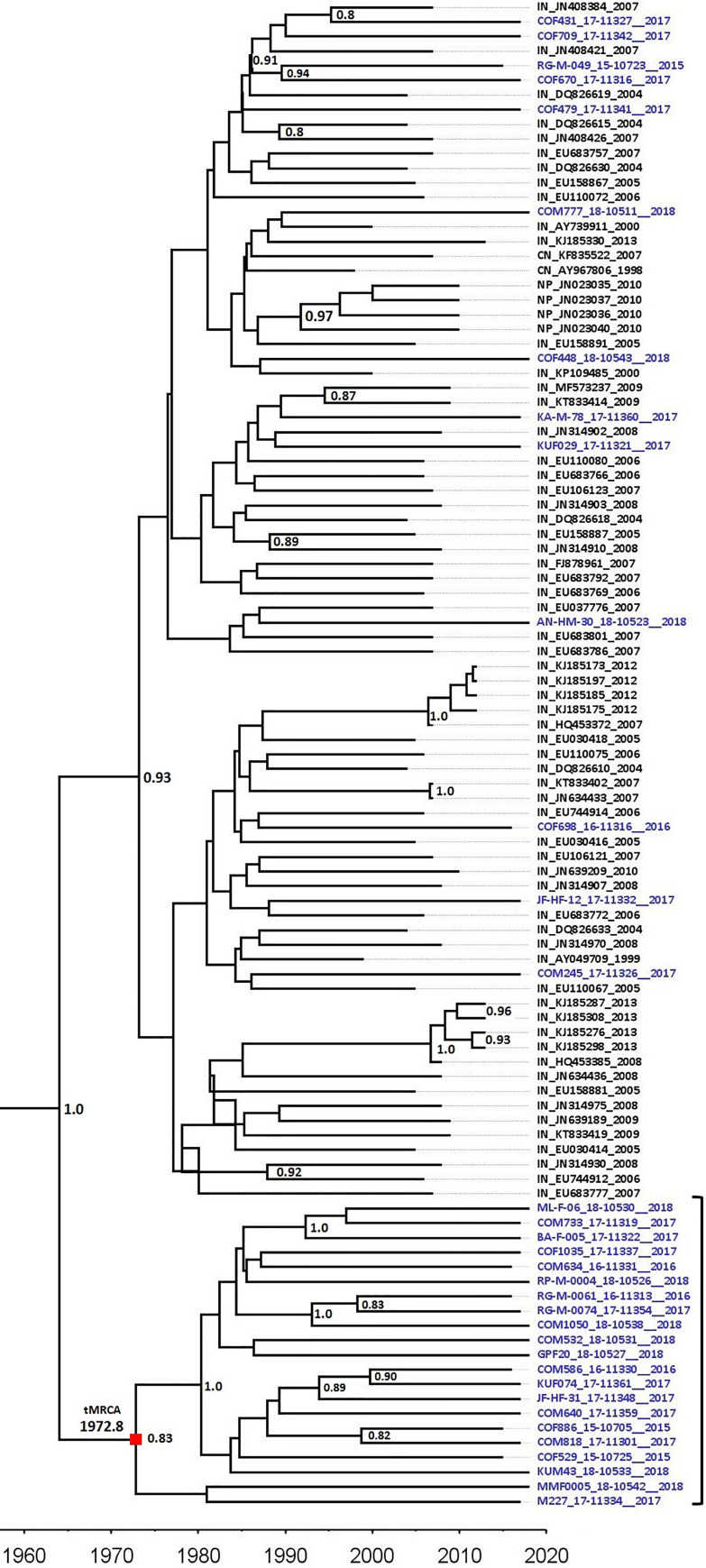
Maximum clade credibility tree obtained after BEAST analysis. Taxon with blue names represent the Sri Lankan sequences (n = 44). The vertical black bracket indicates the Sri Lankan monophyletic cluster. The red square demonstrates the node representing the tMRCA for the monophyletic cluster. The first two letters in the reference taxon names represent the geographical locations of the sequence (CN = China, IN = India and, NP = Nepal). Posterior probability values of ≥0.8 are displayed.

Tracer analysis of BEAST derived parameters revealed that the effective sampling size (ESS) for most of them was more than 200, indicating a strong support for the observed parameters. The mean rate of substitution per site per year was estimated to be 1.09 X 10^−3^ (95% HPD: 6.10X10-4–1.53 X 10^−3^). As shown in **Fig 5**, the estimates of the tMRCA for Sri Lankan monophyletic node with posterior probability 0.83, highlights that these strains were introduced in Sri Lanka around 1972.8 (95%HPD: 1946.88–1987.14).

**Fig 6:**
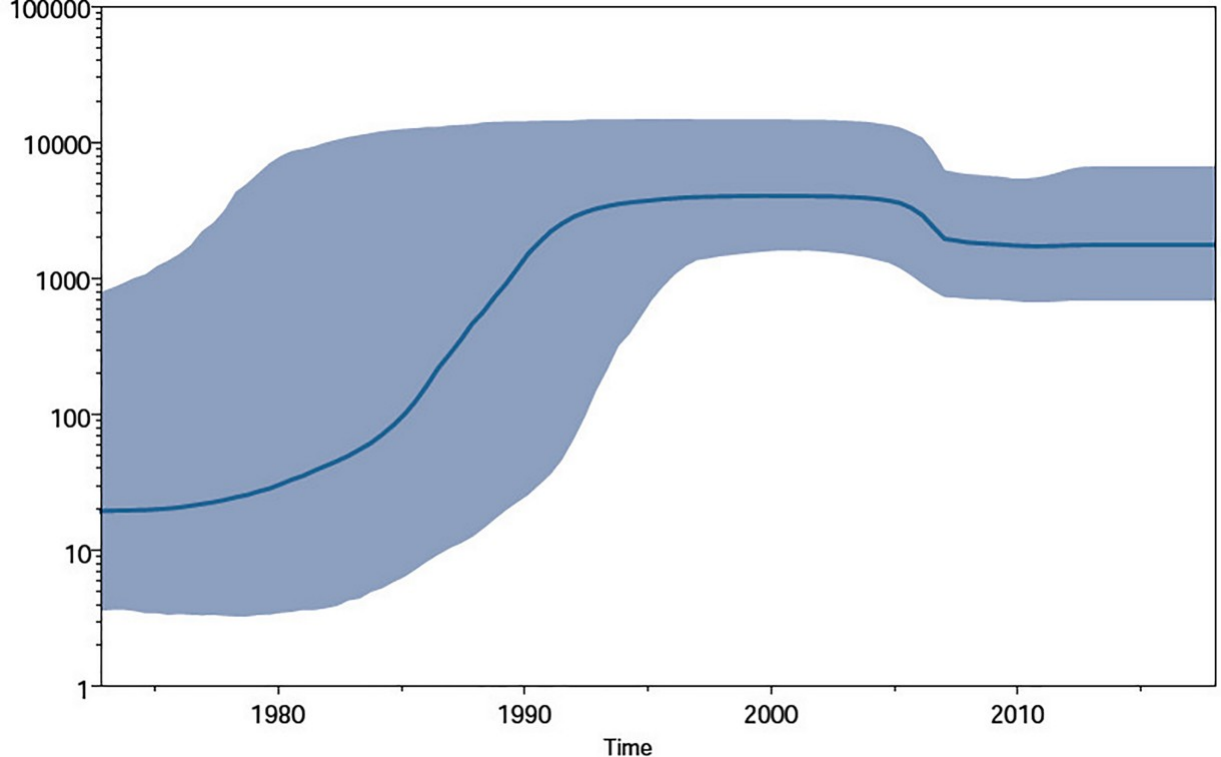
Bayesian Skyline plot obtained using the Sri Lankan HIV-1 subtype C monophyletic cluster. The plot represents the number of effective infections (Y-axis) over time (X-axis). The thick solid line represents the median population and the grey area represents the 95% HPD interval.

Population dynamic estimates were obtained by performing a Bayesian Skyline plot (**Fig 6**) analysis for the Sri Lankan monophyletic cluster. The effective number of infections grew exponentially from the estimated time of introduction (1972.8) until 1990. The trend was observed to be stationary after 1990 until 2005, and between 2005 and 2008 the effective number of infections went down. After 2008, the effective number of infections maintained a stationary phase until 2018.

## Discussion

The first case of HIV infection in Sri Lanka was reported in 1987 [25]. Although the HIV epidemic in Sri Lanka has been categorized as a low-level epidemic, trends in the reported HIV infections have seen an upward trend since 1987 [3]. The HIV epidemic in the country is well tracked with respect to HIV prevalence, transmission dynamics, and key population size via programs like HIV Sentinel Surveillance (HSS) and Integrated Biological & Behavioral Surveillance system (IBBS) [3, 26]. While the surveillance of HIV-1 molecular epidemiology is of paramount importance in terms of vaccine design and testing strategies [10], the information with respect to HIV-1 subtypes in Sri Lanka is still lacking. In this report, using *pol* gene sequence data obtained from HIV-1 infected individuals in Sri Lanka, we are highlighting the presence of HIV-1 subtypes in Sri Lanka. Out of 85 sequences obtained, 44 sequences (51.8%) were identified as subtype C, indicating the dominance of this subtype. Other than subtype C, the sequences categorized as pure subtypes were mainly of subtype B (12.9%), followed by sequences classified as subtypes A1, G, and subtype D. In addition to pure subtypes, there were 15 sequences which were observed to be recombinants. These recombinant sequences mainly constituted CRFs like CRF01_AE, CRF02_AG, and other recombinant sequences (BC, A1D, A1C, and CRF01_AEB recombinant; n = 10) which could be potential URFs. Overall, these findings highlight the diverse nature of HIV-1 subtypes in Sri Lanka.

HIV-1 subtype C is the dominant subtype in Southern Africa, Ethiopia, and India [10]. The HIV-1 subtype C epidemic in India was observed to be the oldest HIV-1 subtype C epidemic outside Africa [27]. While most of the Sri Lankan sequences were observed to be subtype C, our estimates of HIV-1 subtype C tMRCA for Sri Lanka allude to the introduction of this subtype in Sri Lanka from India during the early 1970s (1972.8). However, the major limitation of our analysis is that the number of sequences used for tMRCA estimation is limited and that they represent only a small region of the HIV-1 genome. In addition, there were few Sri Lankan HIV-1 subtype C sequences that were not considered for evolutionary analysis were clustering with reference sequences of African origin, indicating the multiple geographical sources of introduction of HIV-1 subtype C in Sri Lanka. Hence, these observations should be looked into with caution, as analysis with a larger number of sequences and inclusion of sequences clustering with African reference sequences may affect the estimates of the introduction of HIV-1 subtype C in Sri Lanka.

Population dynamics estimates using Sri Lankan subtype C *pol* gene sequences indicate that the HIV-1 subtype C epidemic in Sri Lanka experienced an exponential growth between 1972.8 and 1990, while it maintained a static phase from 1990 until 2005. A brief period between 2005 and 2008 saw a decline in the number of infections, while the epidemic maintained a steady state between 2008 and 2018. For most of the period between 1990 and 2018, our estimates for the population infected with subtype C are in line with the estimates of PLHIVs in Sri Lanka [2]. However, there are subtle differences for estimates between 2005 and 2018, which may owe to the fact that our Bayesian skyline estimates are limited to subtype C, while the PLHIV estimates for Sri Lanka takes in to account all of the HIV infected population irrespective of subtype. Nevertheless, the observed concordance could be attributed to the possibility of subtype C being the dominant subtype, as is the case in our observations too, but not the only HIV-1 subtype present in the country.

The free ART program in Sri Lanka was initiated in 2004 and since 2016, the country has adhered to the test and treat policy. On its path to the 90-90-90 target, Sri Lanka had 1574 (58%) PLHIVs on ART at the end of 2018. ART roll-out programs have been beneficial in achieving low morbidity and mortality in low-middle income countries (LMIC). However, the increasing use of ART has led to the problem of acquired and transmitted drug resistance [28]. HIV-1 DRM analysis for Sri Lankan sequences showed that 65.9% of these sequences had the DRMs against NRTIs, NNRTIs, or both the drug classes. The frequency of M184V and K103N DRMs was the highest in terms of resistance mutations against NRTIs and NNRTIs, respectively.

HIV-1 subtype diversity and occurrence of recombinants pose a challenge in terms of the emergence of drug resistance mutations, virus adaptation to the host, and drug pressure. Moreover, genetic variability may affect the rate of disease progression [29–31]. Even though, the dominance of subtype C sequences hints towards the HIV-1 subtype C being the major subtype in Sri Lanka, the presence of non-subtype C and recombinant sequences cannot be ignored. Considering the nature of challenges presented by HIV-1 diversity, further molecular surveillance of HIV-1 in Sri Lanka with analyses of multiple gene regions or full-length genomes may provide better insights into HIV-1 molecular epidemiology from this part of the world.

## Supporting information

S1 Table Primers used for performing amplification. (DOCX)

S2 Table Patient demographic characteristics. (DOCX)

S3 Table HIV-1 subtyping results. (DOCX)
