# Supplementary material for: HIV-1 pol gene diversity and molecular dating of subtype C from Sri Lanka
Source: PLoS One. 2020 Jun 11;15(6):e0234133. doi: 10.1371/journal.pone.0234133 (PMC7289380; doi:10.1371/journal.pone.0234133)
Supplement: S1 Table — (DOCX) [file pone.0234133.s001.docx]

S1. Table. Primers used for performing amplification

| **Primer** | **Sequence** | **HXB2 coordinates** |
| --- | --- | --- |
| PR1 | TGAARGAITGYACTGARAGRCAGGCTAAT | 2057 → 2085 |
| PR2 | AYCTIATYCCTGGTGTYTCATTRTT | 2979 → 2955 |
| RT1 | TTTYAGRGARCTYAATAARAGAACTCA | 2777 → 2803 |
| RT2 | CCTCITTYTTGCATAYTTYCCTGTT | 3623 → 3599 |
| PR3 | YTCAGRCAGRCCRGARCCAACAGC | 2135 → 2159 |
| PR4 | CTGGTGTYTCATTRTTKRTACTAGGT | 2970 → 2945 |
| RT3 | TTYTGGGARGTYCARYTAGGRATACC | 2808 → 2833 |
| RT4 | GGYTCTTGRTAAATTTGRTATGTCCA | 3583 → 3558 |
