# Supplementary material for: HIV-1 pol gene diversity and molecular dating of subtype C from Sri Lanka
Source: PLoS One. 2020 Jun 11;15(6):e0234133. doi: 10.1371/journal.pone.0234133 (PMC7289380; doi:10.1371/journal.pone.0234133)
Supplement: S2 Table — (DOCX) [file pone.0234133.s002.docx]

S2. Table. Patient demographic characteristics

| Characteristic | Categories | No. (% / Range) |
| --- | --- | --- |
| Gender | Male | 46 (54.1%) |
|  | Female | 39 (45.9%) |
|  |  |  |
| Area | Anuradhapura | 03 (3.53%) |
|  | Badulla | 04 (4.71%) |
|  | Batticaloa | 01 (1.71%) |
|  | Colombo | 11 (12.94%) |
|  | Galle | 03 (3.93%) |
|  | Gampaha | 19 (22.35%) |
|  | Jaffna | 04 (4.71%) |
|  | Kalutara | 07 (8.24%) |
|  | Kandy | 01 (1.71%) |
|  | Kegalle | 05 (5.88%) |
|  | Kurunegala | 06 (7.06%) |
|  | Matale | 02 (2.35%) |
|  | Matara | 03 (3.53%) |
|  | Monaragala | 02 (2.35%) |
|  | Polonnaruwa | 02 (2.35%) |
|  | Puttalam | 07 (8.24%) |
|  | Ratnapura | 01 (1.71%) |
|  | Trincomalee | 01 (1.71%) |
|  | Not known | 03 (3.53%) |
|  |  |  |
| Median Age (Years)^#^ |  | 40 (03-70) |
|  |  |  |
| Median Viral Load (Copies/ML)^@^ |  | 46070 (234-3471621) |
|  |  |  |
| ART Status | Treated | 78 (89.4%) |
|  | Not known | 07 (10.6%) |
|  |  |  |
| Regimen Type | ABC/3TC/EFV | 01 (1.2%) |
|  | ABC/3TC/LPV | 03 (3.5%) |
|  | AZT/3TC/EFV | 10 (11.8%) |
|  | AZT/3TC/LPV | 03 (3.5%) |
|  | AZT/3TC/ATV | 01 (1.2%) |
|  | AZT/3TC/DRV | 01 (1.2%) |
|  | AZT/3TC/NVP | 03 (3.5%) |
|  | AZT/3TC/RAL | 01 (1.2%) |
|  | AZT/3TC/RAL/ATV | 01 (1.2%) |
|  | TDF/FTC/EFV | 36 (42.4%) |
|  | TDF/FTC/LPV | 10 (11.8%) |
|  | TDF/FTC/RAL | 03 (3.5%) |
|  | TDF/FTC/ATV | 03 (3.5%) |
|  | Not known | 09 (10.6%) |
|  |  |  |
| Travel history outside Sri Lanka |  | 26 (30.6%) |

**S2. Table**. # - Data for 84 patients as no data was available for one patient, @ - Data for 79 patients as no data was available for 6 patients. Regimen type - Abacavir (ABC), emtricitabine (FTC), lamivudine (3TC), tenofovir (TDF), zidovudine (AZT), efavirenz (EFV), nevirapine (NVP), atazanavir (ATV), darunavir (DRV), lopinavir (LPV) and raltegravir (RAL)
