# Supplementary material for: HIV-1 pol gene diversity and molecular dating of subtype C from Sri Lanka
Source: PLoS One. 2020 Jun 11;15(6):e0234133. doi: 10.1371/journal.pone.0234133 (PMC7289380; doi:10.1371/journal.pone.0234133)
Supplement: S3 Table — (DOCX) [file pone.0234133.s003.docx]

S3. Table.HIV-1 subtyping results

| No | Sequence | Subtype |
| --- | --- | --- |
| 1 | STD-CHM-125-18_18-10529 | 01_AE |
| 2 | KUM59_18-10532 | 01_AE |
| 3 | NG-F-001_18-10535 | 01_AE |
| 4 | COM568_16-11321 | 01_AE |
| 5 | JF-HM-18_17-11351 | 01_AEB |
| 6 | MMF0002_18-10540 | A1 |
| 7 | KUM47_16-11319 | A1 |
| 8 | KT-M-0005_16-11328 | A1 |
| 9 | KT-F-0006_16-11304 | A1 |
| 10 | COF1306_18-10544 | A1 |
| 11 | COF111_16-11308 | A1 |
| 12 | COF876_18-10534 | A1C |
| 13 | KB-M-005_18-10528 | A1D |
| 14 | KB-M-005_16-11334 | A1D |
| 15 | F875_16-11306 | A1D |
| 16 | COF590_17-11350 | A1D |
| 17 | COF590_15-10726 | A1D |
| 18 | IDM35_17-11331 | 02_AG |
| 19 | IDF576_15-10720 | G |
| 20 | RG-M-0174_17-11356 | B |
| 21 | RG-M-0064_16-11327 | B |
| 22 | KT-M-R-86-17_17-11340 | B |
| 23 | KBM36_17-11335 | B |
| 24 | JFHF01_17-11362 | B |
| 25 | IDM43_15-10719 | B |
| 26 | IDF24_17-11317 | B |
| 27 | COM853_18-10537 | B |
| 28 | COM453_16-11307 | B |
| 29 | COM271_15-10707 | B |
| 30 | COF727_17-11324 | B |
| 31 | MMM0003_18-10541 | BC |
| 32 | KT-F-13_17-11336 | BC |
| 33 | COM453_18-10509 | BC |
| 34 | RP-M-0004_18-10526 | C |
| 35 | RG-M-049_15-10723 | C |
| 36 | RG-M-0074_17-11354 | C |
| 37 | RG-M-0061_16-11313 | C |
| 38 | MMF0005_18-10542 | C |
| 39 | ML-F-06_18-10530 | C |
| 40 | M359_15-10724 | C |
| 41 | M227_17-11334 | C |
| 42 | KUM43_18-10533 | C |
| 43 | KUM-0045_17-11358 | C |
| 44 | KUF074_17-11361 | C |
| 45 | KUF029_17-11321 | C |
| 46 | KA-M-78_17-11360 | C |
| 47 | KA-F-79_17-11345 | C |
| 48 | KA-F-79_16-11303 | C |
| 49 | KA-F-74_16-11332 | C |
| 50 | JF-HF-31_17-11348 | C |
| 51 | JF-HF-12_17-11332 | C |
| 52 | GPF20_18-10527 | C |
| 53 | COM818_17-11301 | C |
| 54 | COM777_18-10511 | C |
| 55 | COM733_17-11319 | C |
| 56 | COM640_17-11359 | C |
| 57 | COM634_16-11331 | C |
| 58 | COM586_16-11330 | C |
| 59 | COM532_18-10531 | C |
| 60 | COM504_17-11303 | C |
| 61 | CO-M-451_16-11305 | C |
| 62 | COM245_17-11326 | C |
| 63 | COM1050_18-10538 | C |
| 64 | COM1010_17-11302 | C |
| 65 | COM_304_16-11309 | C |
| 66 | COF886_15-10705 | C |
| 67 | COF709_17-11342 | C |
| 68 | COF698_16-11316 | C |
| 69 | COF670_17-11316 | C |
| 70 | COF529_15-10725 | C |
| 71 | COF479_17-11341 | C |
| 72 | COF458_15-10702 | C |
| 73 | COF448_18-10543 | C |
| 74 | COF431_17-11327 | C |
| 75 | COF1035_17-11337 | C |
| 76 | BA-F-005_17-11322 | C |
| 77 | AN-HM-30_18-10523 | C |
| 78 | RG-M-0007_15-10722 | D |
| 79 | RGF127_16-11311 | D |
| 80 | F-0229-17-KT_17-11343 | D |
| 81 | KUM-091_17-11357 | G |
| 82 | KUM089_18-10514 | G |
| 83 | KT-M-011_16-11301 | G |
| 84 | KTF020_17-11318 | G |
| 85 | COF427_18-10539 | G |
